# Role of Phosphatidylinositol 3-Kinase (PI3K), Mitogen-Activated Protein Kinase (MAPK), and Protein Kinase C (PKC) in Calcium Signaling Pathways Linked to the α_1_-Adrenoceptor in Resistance Arteries

**DOI:** 10.3389/fphys.2019.00055

**Published:** 2019-02-06

**Authors:** Alejandro Gutiérrez, Cristina Contreras, Ana Sánchez, Dolores Prieto

**Affiliations:** Departamento de Fisiología, Facultad de Farmacia, Universidad Complutense de Madrid, Madrid, Spain

**Keywords:** ERK-MAPK, PI3K, PKC, L-type Ca^2+^ channel, RyR, intracellular Ca^2+^ mobilization, α_1_-adrenergic vasoconstriction, resistance arteries

## Abstract

Insulin resistance plays a key role in the pathogenesis of type 2 diabetes and is also related to other health problems like obesity, hypertension, and metabolic syndrome. Imbalance between insulin vascular actions via the phosphatidylinositol 3-Kinase (PI3K) and the mitogen activated protein kinase (MAPK) signaling pathways during insulin resistant states results in impaired endothelial PI3K/eNOS- and augmented MAPK/endothelin 1 pathways leading to endothelial dysfunction and abnormal vasoconstriction. The role of PI3K, MAPK, and protein kinase C (PKC) in Ca^2+^ handling of resistance arteries involved in blood pressure regulation is poorly understood. Therefore, we assessed here whether PI3K, MAPK, and PKC play a role in the Ca^2+^ signaling pathways linked to adrenergic vasoconstriction in resistance arteries. Simultaneous measurements of intracellular calcium concentration ([Ca^2+^]_i_) in vascular smooth muscle (VSM) and tension were performed in endothelium-denuded branches of mesenteric arteries from Wistar rats mounted in a microvascular myographs. Responses to CaCl_2_ were assessed in arteries activated with phenylephrine (PE) and kept in Ca^2+^-free solution, in the absence and presence of the selective antagonist of L-type Ca^2+^ channels nifedipine, cyclopiazonic acid (CPA) to block sarcoplasmic reticulum (SR) intracellular Ca^2+^ release or specific inhibitors of PI3K, ERK-MAPK, or PKC. Activation of α_1_-adrenoceptors with PE stimulated both intracellular Ca^2+^ mobilization and Ca^2+^ entry along with contraction in resistance arteries. Both [Ca^2+^]_i_ and contractile responses were inhibited by nifedipine while CPA abolished intracellular Ca^2+^ mobilization and modestly reduced Ca^2+^ entry suggesting that α_1_-adrenergic vasoconstriction is largely dependent Ca^2+^ influx through L-type Ca^2+^ channel and to a lesser extent through store-operated Ca^2+^ channels. Inhibition of ERK-MAPK did not alter intracellular Ca^2+^ mobilization but largely reduced L-type Ca^2+^ entry elicited by PE without altering vasoconstriction. The PI3K blocker LY-294002 moderately reduced intracellular Ca^2+^ release, Ca^2+^ entry and contraction induced by the α_1_-adrenoceptor agonist, while PKC inhibition decreased PE-elicited Ca^2+^ entry and to a lesser extent contraction without affecting intracellular Ca^2+^ mobilization. Under conditions of ryanodine receptor (RyR) blockade to inhibit Ca^2+^-induced Ca^2+^-release (CICR), inhibitors of PI3K, ERK-MAPK, or PKC significantly reduced [Ca^2+^]_i_ increases but not contraction elicited by high K^+^ depolarization suggesting an activation of L-type Ca^2+^ entry in VSM independent of RyR. In summary, our results demonstrate that PI3K, ERK-MAPK, and PKC regulate Ca^2+^ handling coupled to the α_1_-adrenoceptor in VSM of resistance arteries and related to both contractile and non-contractile functions. These kinases represent potential pharmacological targets in pathologies associated to vascular dysfunction and abnormal Ca^2+^ handling such as obesity, hypertension and diabetes mellitus, in which these signaling pathways are profoundly impaired.

## Introduction

Insulin resistance plays a key role in the pathogenesis of type 2 diabetes and is also associated to other metabolic and cardiovascular abnormalities such as obesity, dyslipidemia and hypertension, jointly referred as to metabolic syndrome (Ford, 2005). Imbalance between insulin vascular actions via the phosphatidylinositol 3-Kinase (PI3K) and the mitogen activated protein kinase (MAPK) signaling pathways in insulin resistant states results in impaired endothelial vasodilator PI3K/eNOS/NO and augmented vasoconstrictor MAPK/endothelin 1 (ET1) pathways leading to endothelial dysfunction and exacerbated vasoconstriction (Kim et al., 2006; Prieto et al., 2013). However, altered Ca^2+^ homeostasis in the arterial wall usually underlies abnormal vasoconstriction and the vascular complications associated to metabolic disease, such as hypertension and coronary artery disease (Okon et al., 2005; Ford et al., 2010; Villalba et al., 2011).

Vascular smooth muscle (VSM) contraction is triggered by the elevation of free intracellular Ca^2+^ concentration [Ca^2+^]_i_ due to extracellular Ca^2+^ influx and/or Ca^2+^ release from intracellular stores in the sarcoplasmic reticulum (SR), followed by Ca^2+^-calmodulin-dependent activation of myosin light chain (MLC) kinase (MLCK), MLC phosphorylation and actin/myosin crossbridges formation (Liu and Khalil, 2018). However, increases in force development at a given cytosolic Ca^2+^ concentration can also occur and hence a dissociation between [Ca^2+^]_i_, MLC phosphorylation and vasoconstriction mediated by Ca^2+^ sensitization mechanisms (Somlyo and Somlyo, 2003; Villalba et al., 2007). Ca^2+^ release from SR intracellular stores, Ca^2+^ entry through plasma membrane channels and Ca^2+^ sensitization mechanisms can differentially contribute to VSM contraction depending on the vasoconstrictor agonist and/or vessel size (Nobe and Paul, 2001; Villalba et al., 2007; Kitazawa and Kitazawa, 2012). Furthermore, relative contribution of protein kinases to Ca^2+^ handling coupled to receptor-mediated arterial vasoconstriction has also been reported to be size-dependent and thus, involvement of PKC and Rho kinase (RhoK) increase and decrease, respectively, with decreasing arterial size (Kitazawa and Kitazawa, 2012; Martinsen et al., 2012). Although kinases such as PKC and RhoK have traditionally been associated to Ca^2+^ sensitization mechanisms involved in smooth muscle contraction (Nobe and Paul, 2001; Somlyo and Somlyo, 2003; Villalba et al., 2007), increasing experimental evidence supports a role for protein kinase-mediated regulation of intracellular Ca^2+^ mobilization and Ca^2+^ entry mechanisms in VSM and cardiac myocytes (Ghisdal et al., 2003; Villalba et al., 2008; Smani et al., 2010), and differences between large/conductance and small/resistance arteries concerning the role of various kinases in Ca^2+^ handling have also been demonstrated (Kitazawa and Kitazawa, 2012; Martinsen et al., 2012).

Peripheral small arteries or resistance arteries, whose vasoconstrictor activity is under the sympathetic nervous control, play a key role in blood pressure regulation, hypertension being a common vascular complication in metabolic syndrome and insulin resistant states (Ford, 2005). Both impairment of the signaling pathways including MAPK, PI3K, and PKC in endothelial cells and altered Ca^2+^ handling in VSM have been reported to underlie abnormal vasoconstriction in metabolic disease (Okon et al., 2005; Kim et al., 2006; Villalba et al., 2011; Prieto et al., 2013). Since the role of MAPK, PI3K, and PKC in Ca^2+^ handling of resistance arteries is poorly understood, we assessed here whether these kinases are involved in Ca^2+^ signaling pathways linked to adrenergic vasoconstriction in resistance arteries.

## Materials and Methods

### Animal Model

Animal care and experimental protocols conformed to the European Directive for the Protection of Animals Used for Scientific Purposes (European Union Directive 2010/63/EU) and were also supervised by the Animal Care and Use Committee Complutense University of Madrid. Male Wistar rats were housed at the Pharmacy School animal care facility under controlled suitable environmental conditions of temperature (24°C), lighting (12 h light/12 h dark cycle) and humidity (50–60%), and maintained on standard chow and water *ad libitum*. They were anesthetized with sodium pentobarbital (50 mg/kg, i.p.) and euthanized by decapitation and exsanguination at 12-weeks age.

### Dissection and Mounting of Mesenteric Resistance Arteries

After animals were euthanized, the mesentery was quickly removed and placed on cold physiological saline solution (PSS) of the following composition (mM): NaCl 119, NaHCO_3_ 25, KCl 4.7, KH_2_PO_4_ 1.17, MgSO_4_ 1.18, CaCl_2_ 1.5, ethylenediaminetetraacetic acid 0.027 and glucose 11, continuously gassed with a mixture of 5% CO_2_ /95% O_2_ to maintain pH at 7.4. Mesenteric resistance arteries, third order branches of the superior mesenteric artery, were carefully dissected by removing the surrounding connective and fat tissue. Arterial segments were mounted in parallel in double microvascular myographs (Danish Myo Technology, DMT-Denmark) by inserting two 40 μm tungsten wires and equilibrated for 30 min in PSS at 37°C. The relationship between passive wall tension and internal circumference was determined for each individual artery. The arteries were set to an internal circumference (L_1_) equal to 90% of that given by a transmural pressure of 100 mmHg for a relaxed vessel *in situ*, L_100_ (L_1_ = 0.9 × L_100_) at which tension development is maximal (Mulvany and Halpern, 1977). At the beginning of each experiment, arteries were stimulated twice with (KPSS), similar to PSS except that NaCl was substituted for KCl on an equimolar basis, in order to test vessel viability. The endothelium was mechanically removed by inserting a human hair in the vessel lumen and guiding it back and forwards several times. The absence of functional endothelium was confirmed by lack of the relaxation to acetylcholine (10 μM). Arteries were chemically denervated by incubation with guanethidine (10 μM) for 45 min to inhibit adrenergic nerve endings.

### Simultaneous Measurements of Intracellular Ca^2+^ ([Ca^2+^]_i_) and Tension

Simultaneous measurements of the intracellular calcium concentration ([Ca^2+^]_i_) and tension were performed by FURA-2 AM fluorescence in mesenteric resistance arteries as previously reported (Villalba et al., 2007). Arteries were incubated in the dark with 8 μM Fura-2 AM in PSS for 2 h at 37°C. The myograph chamber was mounted on a Zeiss inverted microscope equipped for dual excitation wavelength fluorimetry (Deltascan, Photon Technology). After loading, arteries were illuminated with alternating 340 and 380 nm light using a monochromator-based system (Deltascan, Photon Technology). Fluorescence emission was detected at 510 nm wavelength. The Ratio (R) F340/F380 was taken as a measure of [Ca^2+^]_i_. At the end of each experiment fluorescence not related to Ca^2+^ was measured by bathing the artery in PSS containing 25 mM MnCl_2_ plus ionomycin (10 μM) to quench Ca^2+^-insensitive signals and the values obtained were subtracted from those obtained throughout the experiment.

### Experimental Procedures for the Functional Experiments

The role of ERK-MAPK, PI3K, and PKC kinases in Ca^2+^ handling of resistance arteries was assessed in endothelium-denuded arteries kept in Ca^2+^-free medium. Arteries were exposed for 5 min to Ca^2+^-free PSS (0 mM Ca^2+^, 0.1 mM EGTA) to remove all extracellular Ca^2+^ available for contraction. The myograph solution was then replaced by “nominally Ca^2+^-free PSS” (0 mM Ca^2+^, 0 mM EGTA) and concentration-response curves (CRCs) for CaCl_2_ (10 μM-3 mM) were performed in arteries activated with phenylephrine (PE, 10 μM), in the absence (controls) and presence of the selective blocker of L-type Ca^2+^ channels nifedipine (0.3 μM), the inhibitor of the Orai1-mediated Ca^2+^ entry Pyr6 (3 μM) or the specific inhibitors of ERK-MAPK (PD-98059, 3 μM), p38MAPK (SB-203580, 0.3 μM), PI3K (LY-294002, 3 μM) or PKC (GF-109203X, 0.1 μM). The effect of SR Ca^2+^ store depletion on Ca^2+^ entry and contraction stimulated by PE was assessed in arteries kept in nominally Ca^2+^-free medium stimulated with 10 μM PE and then activated with a single Ca^2+^ concentration (1 mM), before and after SR Ca^2+^ATPase (SERCA) inhibition with cyclopiazonic acid (CPA, 10 μM), and then treatment with CPA plus nifedipine. The combined effect of SR Ca^2+^ store depletion with CPA (10 μM) and inhibition of Orai1-mediated Ca^2+^ entry channels with Pyr6 (3 μM) was also examined on Ca^2+^ entry and vasoconstriction of mesenteric arteries stimulated with PE (10 μM).

The inhibitory effect of PD-98059 (3 μM), LY-294002 (3 μM), or GF-109203X (0.1 μM) on Ca^2+^ entry was also assessed in KPSS-depolarized arteries. After a first stimulation with KPSS, arteries were incubated with the inhibitors for at least 30 min before a second stimulation with KPSS was repeated. To evaluate the potential relationship between the PI3K, MAPK, and PKC pathways and the ryanodine receptor (RyR)-mediated Ca^2+^-induced Ca^2+^-release (CICR) mechanism in resistance arteries (Sánchez et al., 2018), RyR was blocked by incubation with 10 μM ryanodine for 25 min and then 1.5 mM CaCl_2_ was added to arteries depolarized with Ca^2+^-free high K^+^ solution (KPSS^0^_0_). The effect of the selective inhibitors of ERK-MAPK, PI3K, or PKC was further assessed in arteries under conditions of RyR blockade.

### Solutions and Drugs

Ca^2+^-free PSS and Ca^2+^-free KPSS solutions were similar to PSS and KPSS, respectively, except that CaCl_2_ was replaced by 100 μM of EGTA, which was omitted when CaCl_2_ was administered *(“nominally Ca^2+^-free solution*,” 0 mM Ca^2+^, 0 mM EGTA). Acetylcholine, guanethidine, and phenylephrine were obtained from Sigma-Aldrich (Spain). All of them were dissolved in distilled water. Nifedipine, CPA, Pyr6 and kinase inhibitors (PD-98059, LY-294002, GF-109203X, and SB-203580) were obtained from Tocris Cookson (Bristol, United Kingdom). Stock solutions of Pyr6, PD-98059 and LY-294002 were made in distilled water, and those of CPA Pyr6, ryanodine, SB-203580, and GF-109203X in DMSO and further diluted in water. Nifedipine was initially dissolved in ethanol and further dilutions were made in distilled water.

### Statistical Analysis

Results are expressed as either absolute values (units of R F340/F380 or Nm^-1^ of active tension) or as a percentage of the response to KPSS in each artery, as means ± SEM of 6–10 arteries (one artery from each animal). Arterial sensitivity to agonists was expressed in terms of pEC_50_, that was the negative value of log EC_50_, EC_50_ being the concentration of agonist giving 50% of the maximal response or effect (Emax). Statistically significant differences between means were analyzed by using paired or unpaired Student’s *t*-test where appropriate, or one-way ANOVA followed by Bonferroni’s *post hoc* test for comparisons involving more than two groups. Probability levels lower than 5% (*P* < 0.05) were considered statistically significant. Calculations were made using a standard software package (GraphPad Prism 5.0, GraphPad Software, Inc., San Diego, CA, United States).

## Results

### Ca^2+^ Signaling Mechanisms Coupled to the α_1_-Adrenoceptor in Resistance Arteries

**FIGURE 1:**
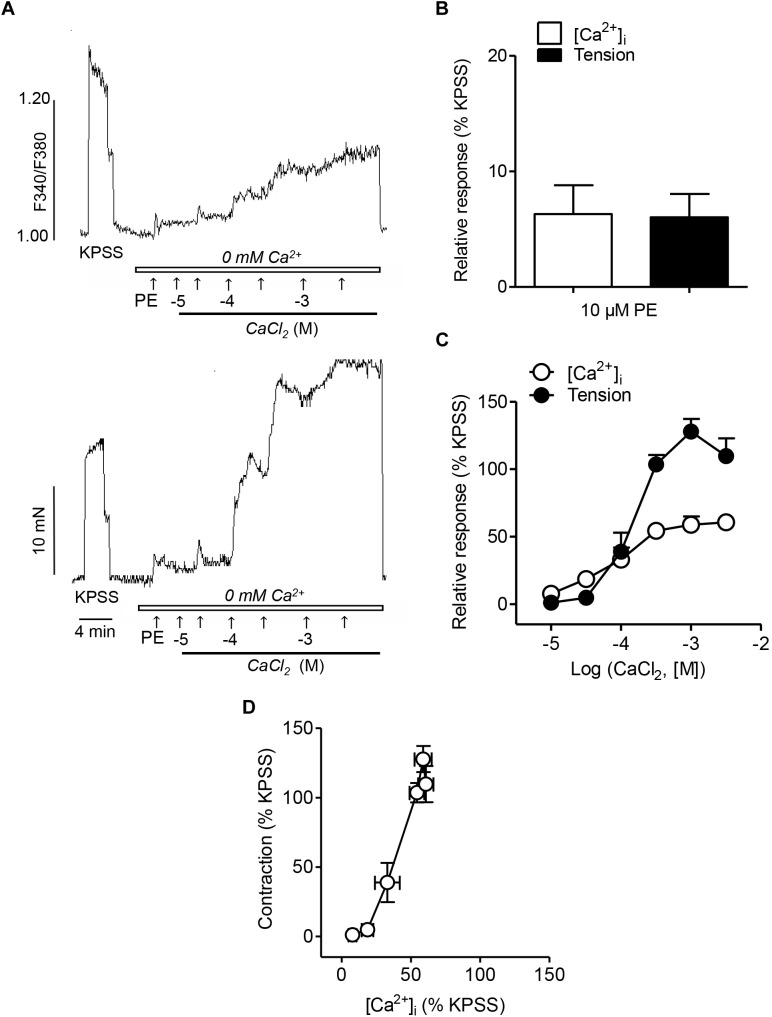
α_1_-Adrenoceptor activation involves intracelular Ca^2+^ mobilization, Ca^2+^ entry and Ca^2+^ sensitization associated to contraction **(A)** Representative traces illustrating the changes in both [Ca^2+^]_i_ mobilization (top) and vasoconstriction (bottom) induced by activation of α_1_-adrenoceptors by a single dose of Phenylephrine (PE) (10 μM) and further cumulative addition of CaCl_2_ (closed bar) in endothelium-denuded mesenteric arteries kept in nominally Ca^2+^-free medium (0 mM Ca^2+^, open bar). **(B,C)** Summarized data showing changes in [Ca^2+^]_i_ and vasoconstrictor responses stimulated by 10 μM PE addition in arteries kept in nominally Ca^2+^-free medium **(B)** and further cumulative re-addition of CaCl_2_ **(C)**. **(D)** Relationship between [Ca^2+^]_i_-contraction in response to cumulative addition of Ca^2+^ in arteries kept in Ca^2+^ free medium and stimulated with 10 μM PE. Responses are expressed as absolute values of either [Ca^2+^]_i_ (ΔF_340/_F_380_) or tension (Nm^-1^) **(A)** or relative to those elicited by KPSS **(B–D)**. Values are means ± SEM of *n* = 7 arteries (one from each animal).

In order to assess the involvement of intracellular Ca^2+^ mobilization and Ca^2+^ entry mechanisms coupled to the α_1_-adrenoceptor in resistance arteries, endothelium-denuded mesenteric arteries were kept in a nominally Ca^2+^-free medium, stimulated with PE and further activated with increasing Ca^2+^ concentrations (Figure 1A). PE induced an initial rapid increase in VSM [Ca^2+^]_i_ and a simultaneous phasic contraction showing intracellular Ca^2+^ mobilization (Figure 1A,C), and a further sustained elevation of [Ca^2+^]_i_ along with vasoconstriction upon Ca^2+^ re-addition, indicative of VSM Ca^2+^ entry (Figure 1A,B). While there were no significant differences in the initial PE-induced [Ca^2+^]_i_ increases and contraction corresponding to intracellular Ca^2+^ mobilization (Figure 1C), PE-induced vasoconstriction upon Ca^2+^ re-addition was larger than the simultaneous sustained [Ca^2+^]_i_ increases (Figure 1C). Involvement of Ca^2+^ sensitization in the α_1_-adrenoceptor-mediated vasoconstriction was further depicted by the steep slope of the [Ca^2+^]_i_ -tension relationship for PE, showing that large contractions are developed without parallel increases in [Ca^2+^]_i_ levels (Figure 1D).

**FIGURE 2:**
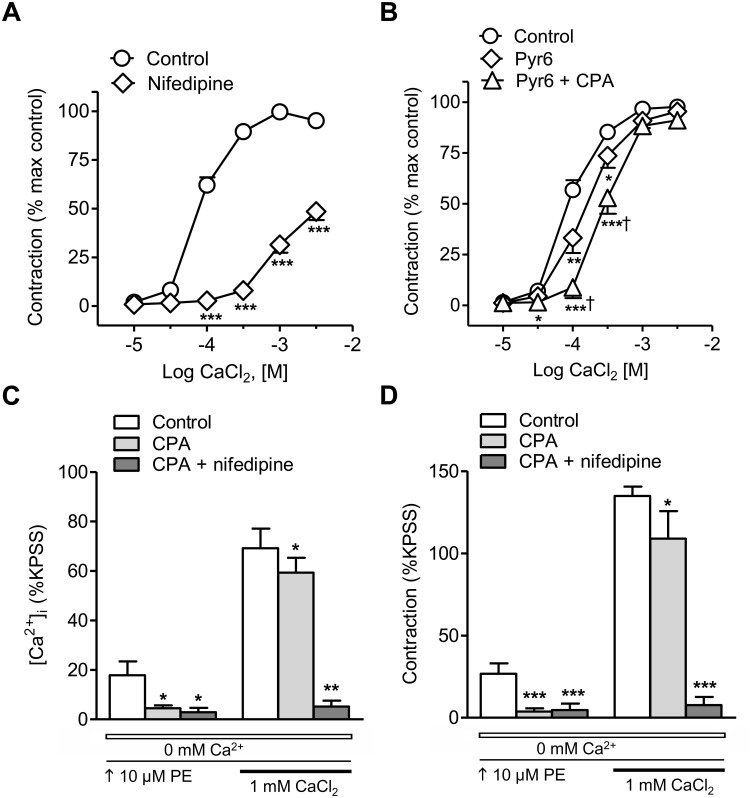
α_1_-Adrenoceptor-elicited vasoconstriction in resistance arteries is mostly due to Ca^2+^ entry through voltage-dependent L-type channels. **(A,B)** Average inhibitory effects of the selective L-type Ca^2+^ channel blocker nifedipine (0.3 μM) **(A)** or the inhibitor of the Orai1–mediated Ca^2+^ entry Pyr6 (3 μM) alone or in combination with the SERCA inhibitor CPA (10 μM) **(B**) on the CaCl_2_ concentration-response curves (CRCs) in endothelium-denuded mesenteric resistance arteries kept in nominally Ca^2+^-free medium and stimulated with 10 μM PE. **(C,D**) Summarized data showing the effects of SR Ca^2+^ store depletion by SERCA inhibition with CPA (10 μM) alone or CPA plus nifedipine (0.3 μM) on the changes in [Ca^2+^]_i_ **(C)** and contraction **(D)** in response to 10 μM PE in resistance arteries kept in a nominally Ca^2+^-free medium (0 mM Ca^2+^, open bar) after Ca^2+^ readmission (1 mM CaCl_2_, closed bar). Results are expressed as a percentage of control maximal responses **(A,B)** or the KPSS responses **(C,D)**. Values are means ± SEM of *n* = 6 arteries (one from each animal). ^∗^*P* < 0.05, ^∗∗^*P* < 0.01, ^∗∗∗^*P* < 0.001 vs. control. ^†^*P* < 0.05 vs. Pyr6-treated.

Treatment with the blocker of L-type voltage-dependent Ca^2+^ channels nifedipine largely inhibited the CaCl_2_ CCR in arteries stimulated with PE (Figure 2A), while the inhibitor of the Orai1-mediated Ca^2+^ entry Pyr6 only induced a moderate decrease of these responses (Figure 2B). Combined treatment with the SERCA inhibitor CPA to deplete SR Ca^2+^ stores plus the inhibitor of store-operated Ca^2+^ channels Pyr6 caused a larger inhibition of PE-induced vasoconstriction (Figure 2B). The effect of SR store depletion by treatment with CPA was further assessed on changes in [Ca^2+^]_i_ and contraction elicited by PE, in arteries kept in nominally Ca^2+^-free medium and further stimulated with 1 mM Ca^2+^ (Figure 2C,D). CPA inhibited PE-induced intracellular Ca^2+^ mobilization and phasic contraction and reduced the sustained Ca^2+^ entry and vasoconstriction elicited by the α_1_-adrenoceptor PE. The latter were abolished by combined treatment with CPA plus the blocker of the L-type voltage-dependent channels nifedipine (Figure 2C,D). These data demonstrate that α_1_-adrenergic vasoconstriction is largely due to Ca^2+^ influx through L-type voltage-dependent Ca^2+^ channels and to lesser extent to Ca^2+^release from the SR and store-operated Ca^2+^ entry.

### PI3K Inhibition Decreased Intracellular Ca^2+^ Mobilization and Ca^2+^ Entry Induced by PE

**FIGURE 3:**
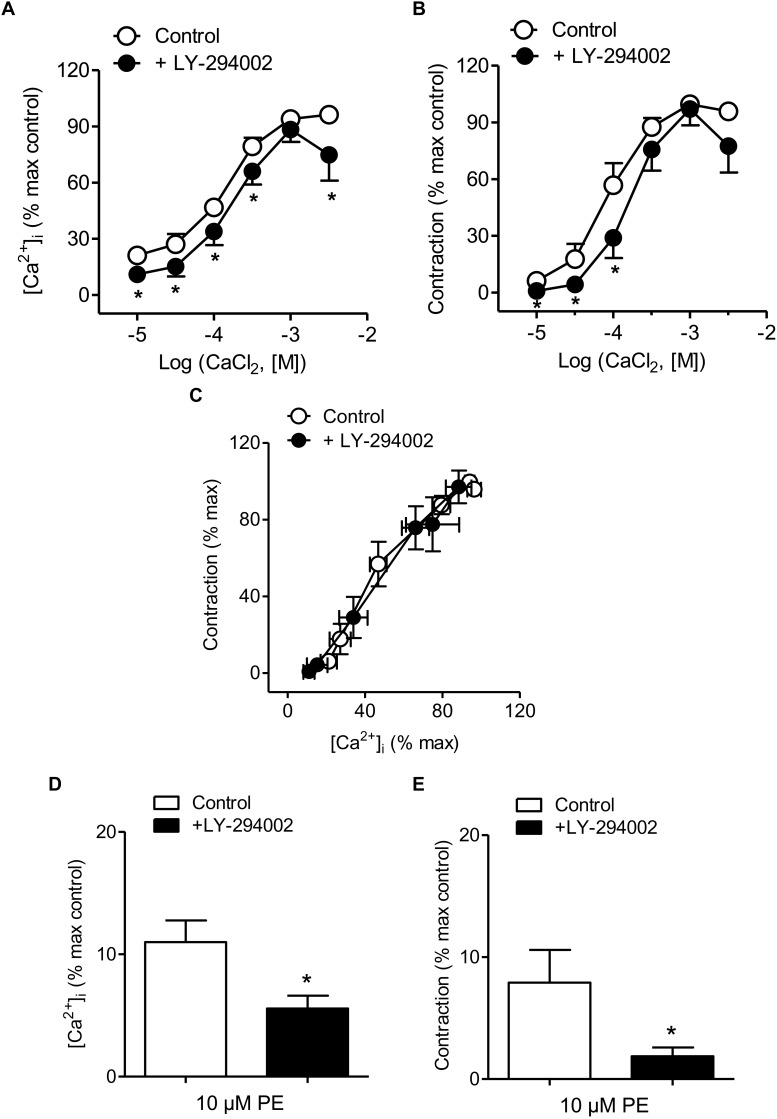
PI3K inhibition reduced Ca^2+^ entry, vasoconstriction and intracellular Ca^2+^ mobilization in response to α_1_-adrenoceptor activation. **(A,B)** Summarized data showing the effects of the PI3K inhibitor LY-294002 (3 μM) on the increases in [Ca^2+^]_i_ **(A)** and contraction **(B)** elicited by cumulative addition of CaCl_2_ in endothelium-denuded arteries kept in a nominally Ca^2+^-free medium and stimulated by 10 μM PE. **(C)** Effect of LY-294002 on the [Ca^2+^]_i_-contraction relationship for 10 μM PE in mesenteric resistance arteries and **(D,E)** the increases in [Ca^2+^]_i_ and contraction elicited by 10 μM PE in nominally Ca^2+^-free PSS. Results are expressed as a percentage of control maximal responses. Values are means ± SEM of *n* = 8 arteries (one from each animal). ^∗^*P* < 0.05 vs. control.

Treatment with the PI3K inhibitor LY-294002 was used to evaluate whether PI3K is involved in Ca^2+^ entry, Ca^2+^ mobilization and/or Ca^2+^ sensitization coupled to the α_1_-adrenoceptor in resistance arteries. This blocker moderately reduced both Ca^2+^ entry and vasoconstriction induced by PE (Figure 3A,B and Table 1), without affecting [Ca^2+^]_i_-contraction relationships for this agonist which suggests no changes in Ca^2+^ sensitization (Figure 3C). Interestingly, PI3K inhibition also reduced PE-induced intracellular Ca^2+^ mobilization and the associated phasic contraction (Figure 3D,E).

**Table 1:** Effects of the inhibitors of PI3K LY-294002 (3 μM), ERK-MAPK kinase PD-98059 (3 μM) and PKC GF-109203X (0.1 μM) on the sensitivity and maximal responses of the CaCl_2_ concentration-response curves in mesenteric arteries stimulated by PE (10 μM) in a Ca^2+^-free medium.

	[Ca^2+^]_i_ (F_340_/F_380_)			Tension (Nm^-1^)		
		
	pEC_50_	E_max_	*n*	pEC_50_	Emax	*n*
Control	3.77 ± 0.06	0.34 ± 0.04	7	4.12 ± 0.09	4.73 ± 0.47	7
+ LY-294002	3.86 ± 0.04	0.25 ± 0.03^∗^	7	3.79 ± 0.09^∗∗^	4.81 ± 0.61	7
Control	3.85 ± 0.08	0.42 ± 0.06	7	3.78 ± 0.06	4.36 ± 0.33	7
+ PD-98059	3.71 ± 0.17	0.23 ± 0.06^∗∗∗^	7	3.73 ± 0.24	4.24 ± 0.32	7
Control	3.96 ± 0.10	0.33 ± 0.04	8	3.91 ± 0.12	5.97 ± 0.63	8
+ GF-109203X	3.90 ± 0.13	0.25 ± 0.04^∗^	8	3.93 ± 0.14	4.81 ± 0.69^∗∗^	8


*Data are means ± SEM; “n” number of arteries (one per animal) from Wistar rats. Results are expressed as absolute values, as the increases in the intracellular Ca^2+^ concentration [Ca^*2*+^]_*i*_ (ratio F_*340*_/F_*380*_) and tension (Nm^-*1*^) elicited by PE or CaCl_*2*_. pEC_*50*_ is – log(EC_*50*_), EC_*50*_ being the concentration of agonist giving 50% of the maximum effect (E_*max*_). Significant differences were analyzed by paired Student’s *t*-test. ^∗^*P* < 0.05; ^∗∗^*P* < 0.01; ^∗∗∗^*P* < 0.001 vs. control.*

### ERK-MAPK Inhibition Reduced Ca^2+^ Entry but Not Vasoconstriction Coupled to the α_1_-Adrenoceptor

**FIGURE 4:**
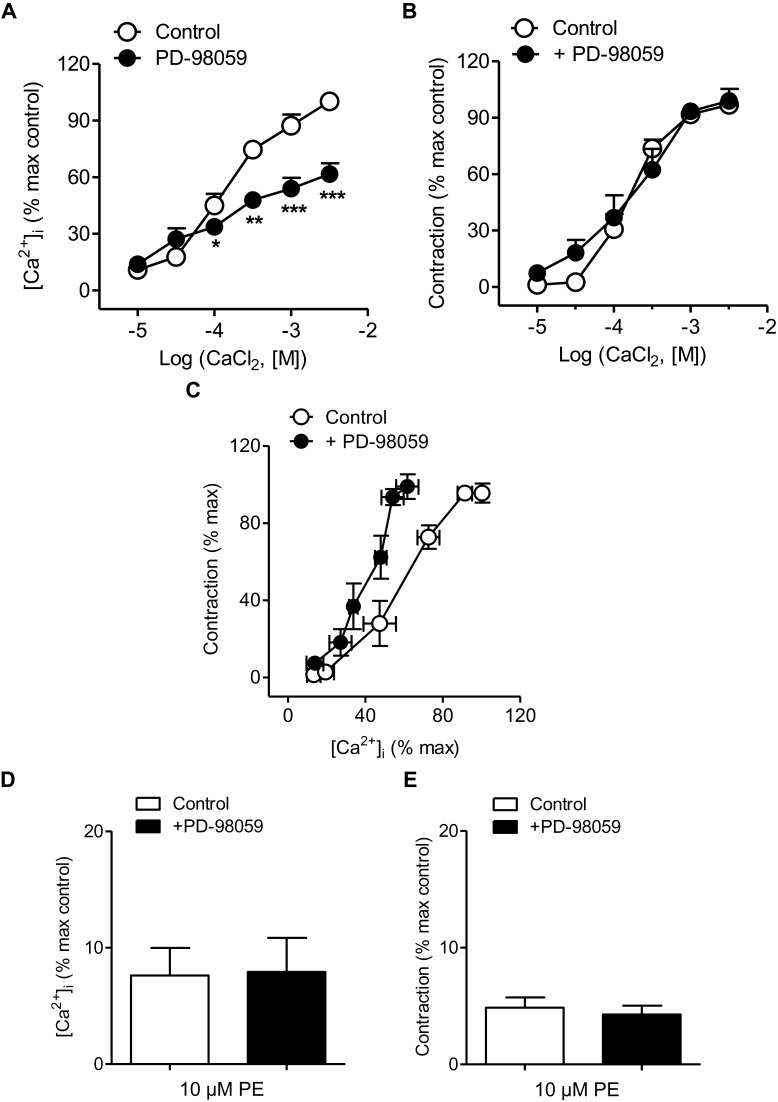
ERK-MAPK inhibition reduced Ca^2+^ entry but not vasoconstriction and increased Ca^2+^ sensitization elicited by α_1_-adrenoceptor activation. **(A,B)** Average effects of the ERK-MAPK inhibitor PD-98059 (3 μM) on the increases in [Ca^2+^]_i_ **(A)** and contraction **(B)** elicited by cumulative addition of CaCl_2_ in endothelium-denuded arteries kept in a nominally Ca^2+^-free medium and stimulated by 10 μM PE. **(C)** Effect of PD-98059 on the [Ca^2+^]_i_-contraction relationship for 10 μM PE in mesenteric resistance arteries and **(D,E)** on the increases in [Ca^2+^]_i_ and contraction elicited by 10 μM PE in Ca^2+^-free PSS. Results are expressed as a percentage of control maximal responses. Values are means ± SEM of *n* = 7 arteries (one from each animal). ^∗^*P* < 0.05, ^∗∗^*P* < 0.01, ^∗∗∗^*P* < 0.001 vs. control.

The effects of the inhibitor of ERK-MAPK PD-98059 on changes in VSM [Ca^2+^]_i_ and contraction in mesenteric resistance arteries kept in nominally Ca^2+^-free medium and activated by PE (10 μM) before increasing CaCl_2_ concentrations were added are shown in Figure 4. Treatment with PD-98059 largely reduced increases in [Ca^2+^]_i_ elicited by PE upon Ca^2+^ re-addition (Figure 4A and Table 1) without altering vasoconstriction (Figure 4B and Table 1). PE-induced contractions were not altered either by treatment with the p38MAPK inhibitor SB-203580 (0.3 μM) (Supplementary Figure S1). The relationship [Ca^2+^]_i_-contraction for PE was left-shifted upon ERK-MAPK kinase blockade in resistance arteries (Figure 4C), indicating decreased Ca^2+^ sensitization under conditions of ERK-MAPK blockade and suggesting Ca^2+^ entry through L-type channels not coupled to vasoconstriction and linked to ERK-MAPK kinase cascade in mesenteric resistance arteries. However, PD-98059 treatment did not affect PE-induced intracellular Ca^2+^ mobilization and contraction associated to the α_1_-adrenergic stimulation (Figure 4D,E).

### PKC Inhibition Reduced Ca^2+^ Entry and Contraction Elicited by PE

**FIGURE 5:**
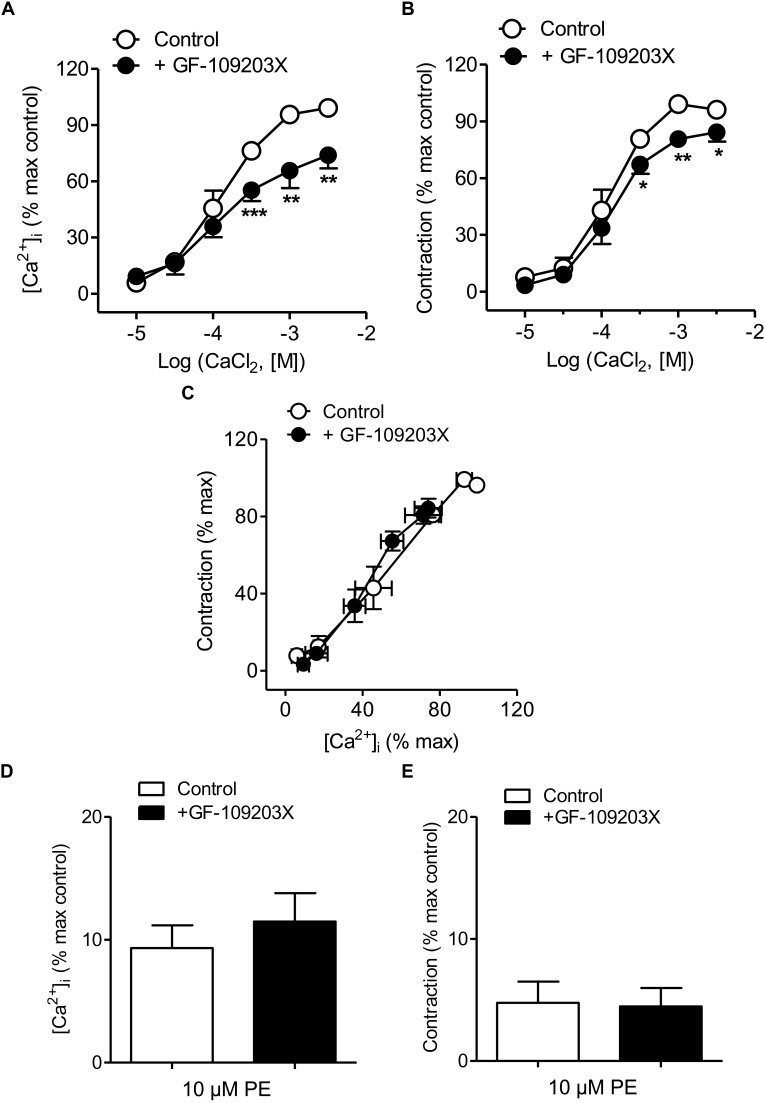
PKC inhibition reduced Ca^2+^ entry and vasoconstriction without changing Ca^2+^ sensitization elicited by α_1_-adrenoceptor activation. **(A,B)** Average effects of the PKC inhibitor GF-109203X (0.1 μM) on the increases in [Ca^2+^]_i_ **(A)** and contraction **(B)** elicited by cumulative addition of CaCl_2_ in endothelium-denuded arteries kept in a Ca^2+^-free medium and stimulated by 10 μM PE. **(C)** Effect of GF-109203X on the [Ca^2+^]_i_-contraction relationships for PE in mesenteric resistance arteries and **(D,E)** on the increases in [Ca^2+^]_i_ and contraction elicited by 10 μM PE in in Ca^2+^-free PSS. Results are expressed as a percentage of control maximal responses. Values are means ± SEM of *n* = 7 arteries (one from each animal). ^∗^*P* < 0.05, ^∗∗^*P* < 0.01, ^∗∗∗^*P* < 0.001 vs. control.

The PKC inhibitor GF-109203X was used to assess the involvement of PKC in Ca^2+^ handling coupled to the α_1_-adrenoceptor in mesenteric resistance arteries. Treatment with GF-109203X reduced the increases in [Ca^2+^]_i_ and to a minor extent vasoconstriction induced by Ca^2+^ re-addition in arteries stimulated by PE kept in a nominally Ca^2+^-free medium (Figure 5A,B and Table 1). Both Ca^2+^ sensitization (Figure 5C) and PE-induced intracellular Ca^2+^mobilization and phasic contraction (Figure 5D,E) remained unaffected by PKC inhibition.

### Inhibition of ERK-MAPK, PI3K, and PKC Pathways Reduced L-Type Ca^2+^ Channel-Mediated [Ca^2+^]_i_ Increases Independently of RyR

Since [Ca^2+^]_i_ changes coupled to the α_1_-adrenoceptor in resistance arteries are largely due to Ca^2+^ entry through L-type Ca^2+^ channels, the effects of PD-98059, LY-294002, and GF-109203X were tested on the increase in [Ca^2+^]_i_ elicited by high K^+^ depolarization in order to assess whether the modulatory effect of ERK-MAPK, PI3K, and PKC on Ca^2+^ entry elicited by PE is exerted through L-type Ca^2+^ channels. Treatment with PD-98059, LY-294002, or GF-109203X reduced Ca^2+^ entry stimulated by KPSS (Supplementary Figures S2A–C) suggesting that these kinases regulate L-type Ca^2+^ channel entry in resistance arteries.

**FIGURE 6:**
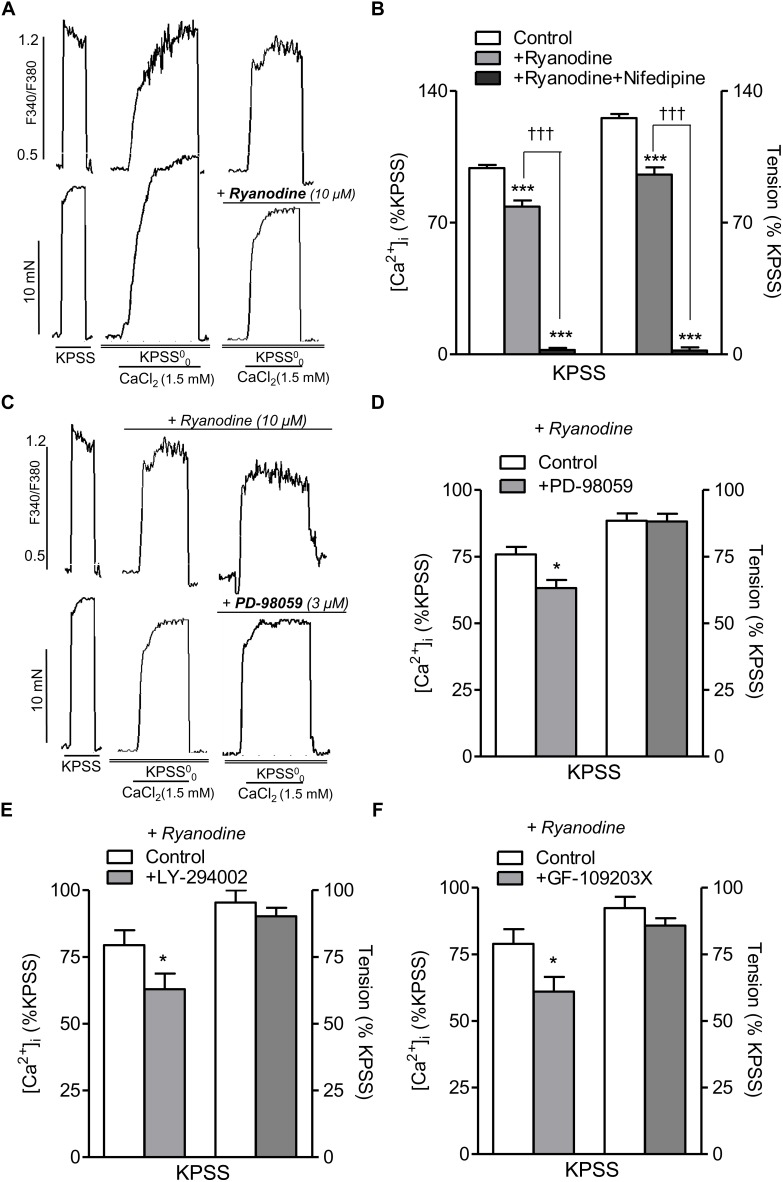
Inhibition of ERK-MAPK, PI3K, or PKC kinase reduces intracelular Ca^2+^ mobilization elicited by voltage-dependent L-type Ca^2+^channel activation independently of RyR-mediated Ca^2+^ release. **(A)** Representative traces showing the effects of ryanodine (10 μM) treatment on changes in both [Ca^2+^]_i_ (top) and vasoconstriction (bottom) induced by CaCl_2_ (1.5 mM) re-addition in endothelium-denuded mesenteric arteries depolarized with high K^+^ in Ca^2+^ free medium (KPSS^0^_0_). **(B)** Summarized data showing the average effects of ryanodine (10 μM) on changes in [Ca^2+^]_i_ and contraction elicited by CaCl_2_ (1.5 mM) re-addition in arteries depolarized with a high K^+^ solution. **(C)** Representative traces showing the effects of inhibition of MAPK with PD-98059 (3 μM) on changes in both [Ca^2+^]_i_ (top) and vasoconstriction (bottom) induced by CaCl_2_ (1.5 mM) re-addition in endothelium-denuded mesenteric arteries depolarized with high K^+^ (KPSS^0^_0_) and treated with ryanodine (30 μM) to block RyR. **(D)** Summarized data showing the average effects of PD-98059 (3 μM) on changes in [Ca^2+^]_i_ and vasoconstriction elicited by CaCl_2_ (1.5 mM) re-addition in arteries depolarized with a high K^+^ solution under conditions of RyR blockade with ryanodine. Average effects of the PI3K inhibitor LY-294002 (3 μM) **(E)** or the PKC inhibitor GF-109203X (0.1 μM) **(F)** on changes in [Ca^2+^]_i_ and vasoconstriction elicited by CaCl_2_ (1.5 mM) re-addition in arteries depolarized with a high K^+^ solution under conditions of RyR blockade. Responses are expressed as absolute values of either [Ca^2+^]_i_ (ΔF_340/_F_380_) or tension (Nm^-1^) **(A,C)** or relative to those elicited by KPSS **(B,D–F)**. Values are means ± SEM of *n* = 5 arteries (one from each animal). Significant differences were analyzed by paired Student’s *t*-test or one-way ANOVA. ^∗^*P* < 0.05 vs. control. ^∗∗∗^*P* < 0.001 vs. control. ^†††^*P* < 0.001 vs. ryanodine-treated.

RyR-mediated Ca^2+^-induced Ca^2+^-release (CICR) upon L-type channel activation has recently been shown in VSM of mesenteric resistance arteries (Sánchez et al., 2018), and therefore we further assessed the potential relationship between PI3K, PKC, and MAPK pathways and RyR-mediated CICR mechanism. Treatment with ryanodine (10 μM) to selectively block the RyR (Meissner, 2017) and the subsequent SR Ca^2+^ mobilization and amplification of Ca^2+^ entry through L-type channels, reduced nifedipine-sensitive increases in [Ca^2+^]_i_ and contraction elicited by Ca^2+^ readdition in high K^+^-depolarized endothelium-denuded arteries (Figure 6A,B), thus confirming CICR upon L-type channel activation. On the other hand, in ryanodine-treated arteries to block CICR, selective inhibition of MAPK with PD-98059 (Figure 6C,D), PI3K with LY-294002 (Figure 6E) or PKC with GF-109203X (Figure 6F) resulted in a further significant reduction of the increases in [Ca^2+^]_i_ but not contraction elicited by Ca^2+^ re-addition in high K^+^-depolarized arteries, thus demonstrating a direct stimulatory effect of MAPK, PI3K, and PKC on L-type Ca^2+^ entry independent of RyR mechanisms.

## Discussion

**FIGURE 7:**
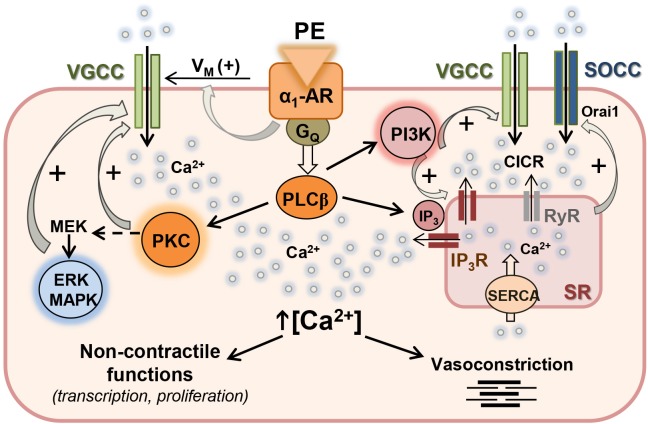
Proposed mechanism for the regulation of Ca^2+^ handling by PI3K, MAPK, and PKC in resistance arteries VSM. Activation of α_1_-adrenoceptor (α_1_-AR) with phenylephrine (PE) is coupled to membrane depolarization (+Vm), stimulation of Ca^2+^ influx through L-type voltage-gated Ca^2+^ channels (VGCC) and vasoconstriction. α_1_-AR is also coupled to PLCβ activation which stimulates Ca^2+^ release from sarcoplasmic reticulum (RS) Ca^2+^ store through IP_3_ receptors (IP_3_R), and also stimulates PKC which in turn activates the MEK-ERK MAPK intracellular signaling cascade. Both PKC and ERK MAPK can directly act on L-type VGCC and reinforce Ca^2+^ influx mostly related to non-contractile functions. α_1_-AR is also coupled to PI3K activation and stimulation of both IP_3_R-mediated Ca^2+^ release and Ca^2+^ entry through VGCC. Ca^2+^ influx through VGCC amplifies Ca^2+^ signal through the ryanodine receptor (RyR)-mediated Ca^2+^-induced Ca^2+^ release (CICR). Emptying of the SR Ca^2+^ store leads to store-operated Ca^2+^ channel (SOCC) entry to refill empty store.

An increasing body of experimental evidence during the last decade gives support to a key role for protein kinase-mediated regulation of Ca^2+^ handling in arterial and cardiac myocytes, through phosphorylation of channels involved in either Ca^2+^ entry through plasma membrane or Ca^2+^ release from SR intracellular stores. The present study provides new insights into the modulation of Ca^2+^ handling by PI3K, MAPK, and PKC signaling pathways coupled to the α_1-_adrenoceptor in resistance arteries and linked to both contractile and non-contractile functions. A graphical summary of our findings is depicted in Figure 7.

Hypertension and augmented vasoconstriction associated to metabolic disease have traditionally been ascribed to protein kinase-mediated Ca^2+^ sensitization of the VSM contractile machinery leading to increased vascular tone and systemic vascular resistance (Martínez et al., 2000; Naik et al., 2006; Villalba et al., 2007, 2011; Crestani et al., 2017). However, Ca^2+^ signaling mechanisms and the relative contribution of Ca^2+^ sensitization and Ca^2+^ mobilization mechanisms to arterial contraction are size-dependent. Thus, sensitivity of vasoconstriction and [Ca^2+^]_i_ changes induced by α_1_-adrenoceptor activation to pharmacological inhibitors of voltage-dependent L-type Ca^2+^ entry is higher in resistance arteries compared to large conductance arteries (Prieto et al., 1991; Kitazawa and Kitazawa, 2012; Martinsen et al., 2012). Moreover, contractions of large arteries have been reported to involve kinases such as RhoK and PKC to varying degrees, while vasoconstriction of resistance arteries seem to be mediated exclusively by PKC (Budzyn et al., 2006; Kitazawa and Kitazawa, 2012). The present results confirm early studies demonstrating the involvement of Ca^2+^ sensitization mechanisms in the noradrenergic vasoconstriction of small mesenteric arteries (Buus et al., 1998; Kitazawa and Kitazawa, 2012), but further demonstrate that PI3K, MAPK, and PKC are involved in the regulation of Ca^2+^ entry and intracellular Ca^2+^ mobilization coupled to α_1_-adrenoceptor activation in resistance arteries.

The PI3K/Akt signaling pathway is stimulated upon activation of the insulin receptor in the vascular endothelium and coupled to eNOS phosphorylation, NO production and vasodilatation. This pathway is impaired and associated to endothelial dysfunction in insulin resistant states (Kim et al., 2006; Contreras et al., 2010). However, PI3Ks play also a main role in the Ca^2+^ signaling of neurons and cardiac and vascular myocytes (Viard et al., 2004; Ghigo et al., 2017). In VSM, PI3Ks have been involved in the regulation of L-type Ca^2+^ entry in response to vasoconstrictors and growth factors (Macrez et al., 2001; Le Blanc et al., 2004) but also in the Ca^2+^-dependent Rho-mediated negative control of MLCP linked to Ca^2+^ sensitization and contraction (Wang et al., 2006). In the present study, pharmacological inhibition of PI3K moderately reduced both Ca^2+^ entry and vasoconstriction elicited by PE in resistance arteries, without affecting the relationship Ca^2+^-tension for the α_1_-adrenergic agonist indicative of changes in Ca^2+^ sensitization. These findings are consistent with earlier studies involving PI3K activation in the transduction pathways for the angiotensin II (AII)-induced Ca^2+^ responses and contraction in VSM (Le Blanc et al., 2004; Rakotoarisoa et al., 2006). In vascular myocytes, PI3Kγ isoform was initially shown to mediate AII-induced activation of L-type voltage-dependent Ca^2+^ currents, to increase [Ca^2+^]_i_ and elicit contraction (Quignard et al., 2001; Le Blanc et al., 2004; Rakotoarisoa et al., 2006). This is confirmed in the endothelium-denuded intact resistance arteries of the present study by the inhibitory effect of the PI3K blocker LY294002 on L-type Ca^2+^ entry elicited by high K^+^ depolarization. PI3Kγ/Akt phosphorylates Ca_v_β_2_ and induces Ca_v_α_1_C translocation thus increasing L-type Ca^2+^ currents in arterial myocytes, and pharmacological inhibition PI3Kγ has vasodilator effects and reduces arterial blood pressure (Carnevale and Lembo, 2012). However, our results further demonstrate that PI3K inhibition markedly reduced intracellular Ca^2+^ release and phasic contraction induced by PE in resistance arteries, which suggests that PI3K might also activate voltage-independent store-operated Ca^2+^ entry through stimulation of intracellular Ca^2+^ release. This pathway is linked to the α_1_-adrenoceptor in resistance arteries, as depicted by the inhibitory effect on Ca^2+^ entry and contraction induced by SR depletion by blockade of SERCA with CPA, or by treatment with the store-operated Ca^2+^ entry blocker Pyr6 (Santiago et al., 2015).

α_1_-Adrenoceptor stimulation causes intracellular Ca^2+^ mobilization via IP_3_ receptor-mediated Ca^2+^ release from SR stores (Liu and Khalil, 2018). Therefore, it seems likely that the reduction of PE-induced intracellular Ca^2+^ mobilization by the PI3K blocker LY294002 to be due to the inhibition of PI3K-stimulated IP_3_-dependent intracellular Ca^2+^ release, as earlier reported for the IGF-1 receptor linked to a G protein-PI3K-phospholipase C signaling pathway in cardiac myocytes (Ibarra et al., 2004). However, we have recently demonstrated RyR-mediated CICR upon activation of L-type channels as a potent amplifying mechanism of Ca^2+^ entry and contraction in mesenteric resistance arteries (Sánchez et al., 2018). Since RyR1 and RyR2 can be phosphorylated by kinases such as PKA leading to SR Ca^2+^ leak (Bovo et al., 2017; Meissner, 2017), we further investigated whether PI3K may regulate RyR-mediated CICR stimulated by L-type Ca^2+^ channel activation. Under conditions of RyR blockade with ryanodine, selective PI3K inhibition with LY294002 significantly reduced increases [Ca^2+^]_i_ but not contraction elicited by high K^+^ depolarization. Although RyR phosphorylation cannot be ruled out, the present finding suggest that PI3K activation mainly stimulates Ca^2+^ entry through direct regulation of L-type Ca^2+^ channels in resistance arteries, as discussed above and reported for isolated vascular myocytes (Le Blanc et al., 2004).

Enhanced activity of PI3K and up-regulation of PI3Kδ have been found to be associated to augmented L-type Ca^2+^ entry in arterial myocytes from rat models of type I diabetes (Pinho et al., 2010) and insulin resistance (Sánchez et al., 2018). While in the former PI3K contributed to enhanced vasoconstriction, in the latter PI3K-mediated increased Ca^2+^ entry compensated for SR Ca^2+^ store dysfunction. In the heart, PI3Kα/Akt signaling is involved in insulin inotropic actions and activates Ca^2+^ currents in microdomains containing L-type Ca^2+^ channels (Lu et al., 2009). This pathway and the corresponding L-type Ca^2+^ entry is defective in insulin-deficient and resistant states which might contribute to the cardiac contractile dysfunction in diabetic cardiomyopathy (Lu et al., 2007; Ghigo et al., 2017).

On the other hand, ERK1/2 MAPK pathway is involved in insulin mitogenic actions but has also been associated to insulin-mediated enhanced vasoconstriction in insulin resistant states (Kim et al., 2006; Contreras et al., 2010; Prieto et al., 2013). In the present study, the selective inhibitor of ERK-MAPK PD98059 did not alter the initial rapid Ca^2+^ increase in response to PE corresponding to intracellular Ca^2+^ mobilization, but caused a profound inhibition of the sustained Ca^2+^ entry stimulated by PE without altering the associated contraction in resistance arteries. These findings differ from earlier studies involving ERK-MAPK in both Ca^2+^-dependent vasoconstriction coupled to the AII receptor in human resistance arteries (Touyz et al., 1999), and in Ca^2+^ sensitization-mediated contraction of coronary artery myocytes linked to the ET1 receptor (Cain et al., 2002). Moreover, the present results differ from reports involving the p38 and JNK limbs of the MAPK pathway in nifedipine-sensitive vasoconstriction linked to α-adrenoceptors in VSM of large arteries (Ok et al., 2011). In contrast, our results suggest a major role of MAPK in the regulation of L-type Ca^2+^ entry coupled to the α_1_-adrenergic adrenoceptor in resistance arteries, supported by the marked reduction elicited by PD98059 on the high K^+^ depolarization-induced Ca^2+^ entry under conditions of RyR receptor blockade. These findings are in agreement with reports showing that ERK MAPK phosphorylates Ca_v_1.2 channels and enhances L-type currents in cardiac myocytes in response to growth factors involved in cardiac hypertrophy (Takahashi et al., 2004). Differences in the involvement of the ERK-MAPK in Ca^2+^ handling and contraction in vascular myocytes may be ascribed to the differential activation of this pathway by various receptors. Our results suggest that ERK-MAPK-mediated modulation of Ca^2+^ handling might be related to VSM non-contractile proliferative pathways coupled to the α_1_-adrenoceptor in resistance arteries (Kudryavtseva et al., 2013). Further studies are needed to elucidate the role of receptor-coupled MAPK pathways as modulators of both L-type and non- L-type Ca^2+^ influx involved in cell proliferation.

In cardiac myocytes, ERK MAPK phosphorylation of Ca_v_1.2 channels and increased L-type currents are activated upstream by PKC linked to activation of G protein-coupled receptors (Smani et al., 2010). The role of PKC in arterial Ca^2+^ handling and vasoconstriction is well-documented, although this kinase has mostly been involved in Ca^2+^ sensitization mechanisms coupled to the α_1_-adrenoceptor in resistance arteries (Buus et al., 1998; Villalba et al., 2007; Kitazawa and Kitazawa, 2012). PKC phosphorylates the phosphoprotein CPI-17, a potent inhibitor of MLCP, rendering it inactive and therefore promoting increased vascular tone (Somlyo and Somlyo, 2003). However, in our study, the PKC inhibitor GF-109203X did not alter the initial Ca^2+^ rise but markedly reduced the sustained [Ca^2+^]_i_ increase in response to PE, and to a lesser extent the simultaneous vasoconstriction of mesenteric resistance arteries, which suggests a major role for PKC in the regulation of Ca^2+^ entry rather than in the enhancement of myofilament Ca^2+^ sensitivity of arterial myocytes, as also supported by the unchanged Ca^2+^-tension relationship for the α_1_-adrenergic agonist in presence of the PKC inhibitor. Involvement of PKC in Ca^2+^ entry in resistance arteries partially agrees with that recently reported by Kitazawa and Kitazawa (2012). However, these authors showed that GF-109203X induced a small reduction of PE-induced Ca^2+^ entry, while it abolished vasoconstriction and reduced phosphorylation of MLC, CPI-17 and MYPT1, supporting a major involvement of PKC in Ca^2+^ sensitization. The discrepancies between our data showing a minor inhibitory effect of GF-109203X on Ca^2+^ sensitization coupled to PE vasoconstriction and those in the study by Kitazawa and Kitazawa (2012) might be ascribed to the fact that different PKC isoforms mediate Ca^2+^ sensitization and Ca^2+^ entry pathways in arterial smooth muscle (Liu and Khalil, 2018). Thus, the conventional Ca^2+^-dependent PKC isoforms (α, -β1, -β2, and -γ) are activated by cytosolic Ca^2+^ and diacylglycerol, and in turn phosphorylate a wide array of substrates implicated in the regulation of Ca^2+^ fluxes; specifically, PKCα has been involved in the regulation of L-type Ca^2+^ entry in both vascular and cardiac myocytes (Yang et al., 2009; Gulia et al., 2013). In contrast, the novel PKC isoforms appear to mediate vasoconstriction coupled to Ca^2+^ sensitizing pathways (Liu and Khalil, 2018). GF-109203X is selective for the conventional PKCα and β1 isoforms but it may also inhibit the novel δ and ε PKC isoforms at the higher micromolar range used in the study of Kitazawa and Kitazawa (2012) and Liu and Khalil (2018).

In the present study, involvement of PKC in the sustained Ca^2+^ entry induced by PE and sensitive to L-type Ca^2+^ channel blockade is further supported by the marked inhibitory effect found for GF-109203X on the L-type channel-mediated Ca^2+^ entry elicited by high K^+^ depolarization, thus suggesting that PKC modulates voltage-dependent L-type Ca^2+^ entry coupled to the α_1_-adrenergic vasoconstriction in resistance arteries, and supporting that PKC-mediated modulation of L-type channels in arterial myocytes (Gulia et al., 2013) contributes not only to myogenic tone but also to agonist-induced vasoconstriction (Cobine et al., 2007; Potts et al., 2012). Activity of both conventional and novel PKC isoforms is chronically enhanced by hyperglycemia, lipotoxicity and oxidative stress, which has been associated to the cardiovascular complications in the insulin resistant states (Geraldes and King, 2010; Turban and Hajduch, 2011). Abnormal PKC activity contributes to the augmented arterial L-type Ca^2+^ entry and enhanced vasoconstriction in diabetic arteries (Ungvari et al., 1999) and arteries from genetically obese rats (Sánchez et al., 2018), and in the latter compensates for SR Ca^2+^ store dysfunction.

## Conclusion

The present findings demonstrate that PI3K, MAPK, and PKC are involved in the regulation of Ca^2+^ entry and intracellular Ca^2+^ mobilization coupled to α_1_-adrenoceptor activation in resistance arteries and mostly associated to non-contractile functions of VSM. Under conditions of vascular disease, vascular myocytes change from a contractile to a synthetic phenotype that is able to proliferate and migrate. The present results showing a protein kinase-mediated regulation of Ca^2+^ handling linked to the α_1_-adrenoceptor in resistance arteries suggest that changes in PI3K, MAPK, and PKC signaling pathways involving enhanced Ca^2+^ mobilization not coupled to contraction, might participate in the changes toward a VSM proliferative phenotype and be involved in vascular remodeling in hypertension and other insulin resistant states. Further studies are needed to elucidate this issue.

## Author Contributions

DP conceived, designed, and discussed the experiments. AG and AS performed the experiments and analyzed the data. AG, AS, and DP wrote the manuscript. AG, CC, and AS contributed to discussion, and edited the manuscript.

## Conflict of Interest Statement

The authors declare that the research was conducted in the absence of any commercial or financial relationships that could be construed as a potential conflict of interest.

## Supplementary Material

Figure S1 p38MAPK inhibition does not alter PE-induced vasososntriction. Average effects of the p38MAPK inhibitor SB-203580 (0.3 μM) on the contractions elicited by cumulative addition of CaCl_2_ in endothelium-denuded arteries kept in a nominally Ca^2+^-free medium and stimulated by 10 μM PE. Results are expressed as a percentage of control maximal responses. Values means ± SEM of *n* = 6 arteries (two from each animal).

Figure S2 ERK-MAPK, PI3K, or PKC kinase inhibitors reduce Ca^2+^ entry through voltage-dependent L-type channels. Average inhibitory effects ERK-MAPK inhibitor PD-98059 (3 μM) **(A)**, the PI3K inhibitor LY-294002 (3 μM) **(B)** or the PKC inhibitor GF-109203X (0.1 μM) **(C)**, on the increases in [Ca^2+^]_i_ elicited by depolarization high K^+^ (KPSS). Results are expressed as absolute values of [Ca^2+^]_i_ (ΔF_340/_F_380_). Values are means ± SEM of five arteries (one from each animal). Significant differences were analyzed by paired Student’s *t*-test; ^∗^*P* < 0.05 vs. control.
