# Supplementary figures and images for: Role of Phosphatidylinositol 3-Kinase (PI3K), Mitogen-Activated Protein Kinase (MAPK), and Protein Kinase C (PKC) in Calcium Signaling Pathways Linked to the α1-Adrenoceptor in Resistance Arteries
Source: Front Physiol. 2019 Feb 6;10:55. doi: 10.3389/fphys.2019.00055 (PMC6372516; doi:10.3389/fphys.2019.00055)

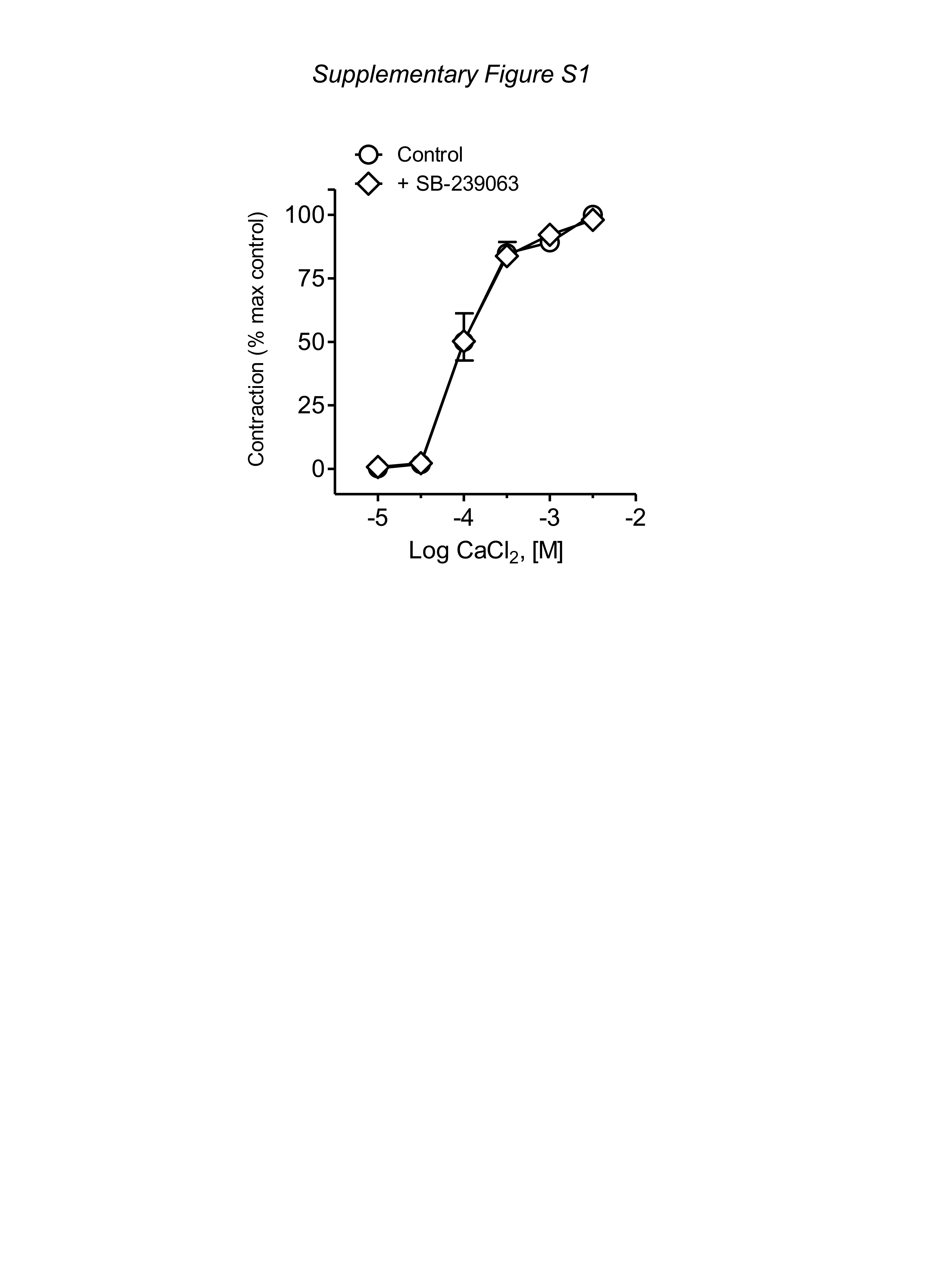

Supplement: Figure S1 — p38MAPK inhibition does not alter PE-induced vasososntriction. Average effects of the p38MAPK inhibitor SB-203580 (0.3 μM) on the contractions elicited by cumulative addition of CaCl2 in endothelium-denuded arteries kept in a nominally Ca2+-free medium and stimulated by 10 μM PE. Results are expressed as a percentage of control maximal responses. Values means ± SEM of n = 6 arteries (two from each animal). [file Image_1.JPEG]

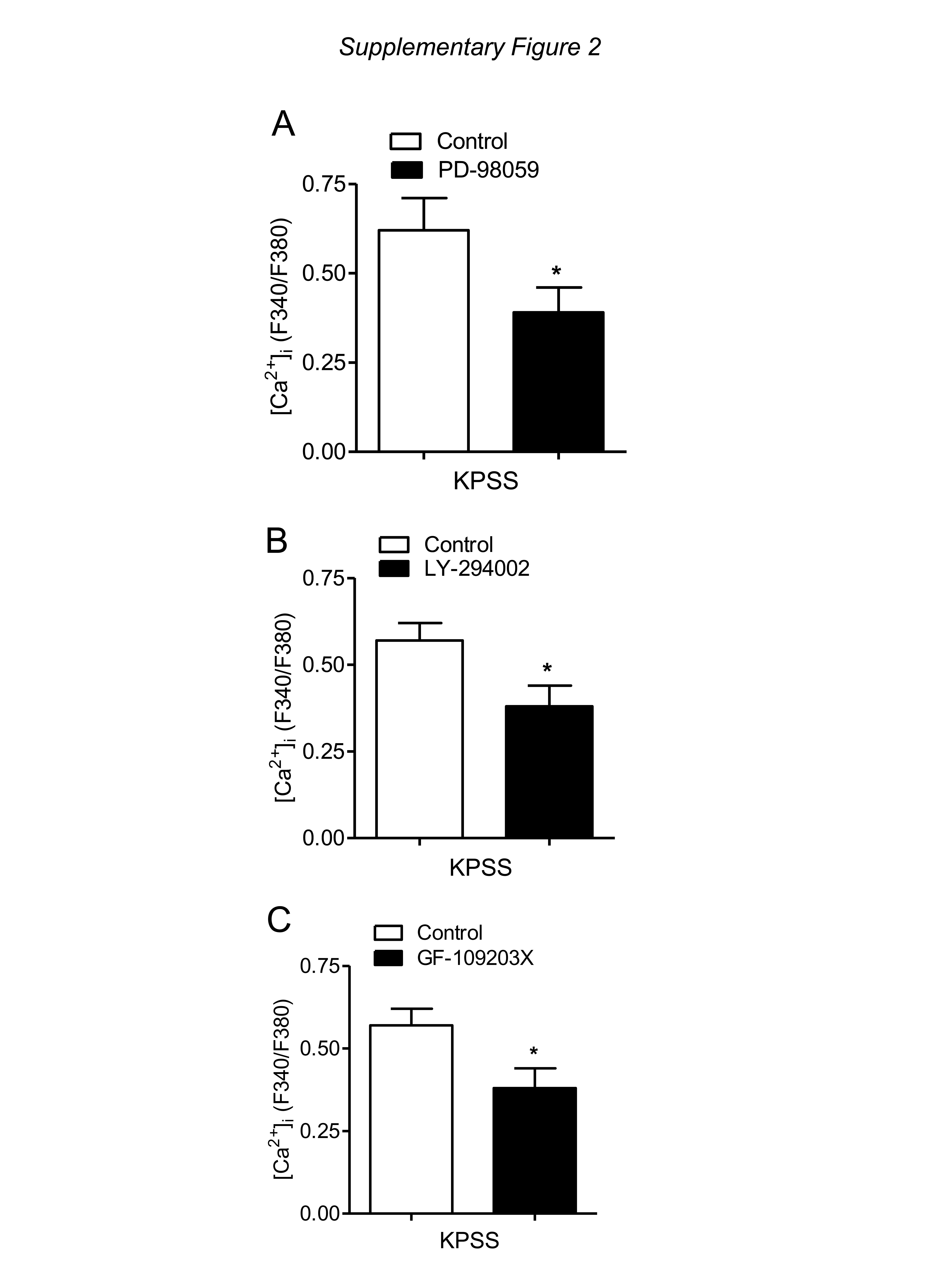

Supplement: Figure S2 — ERK-MAPK, PI3K, or PKC kinase inhibitors reduce Ca2+ entry through voltage-dependent L-type channels. Average inhibitory effects ERK-MAPK inhibitor PD-98059 (3 μM) (A), the PI3K inhibitor LY-294002 (3 μM) (B) or the PKC inhibitor GF-109203X (0.1 μM) (C), on the increases in [Ca2+]i elicited by depolarization high K+ (KPSS). Results are expressed as absolute values of [Ca2+]i (ΔF340/F380). Values are means ± SEM of five arteries (one from each animal). Significant differences were analyzed by paired Student’s t-test; ∗P < 0.05 vs. control. [file Image_2.JPEG]
